# Metabolomic profile of systemic sclerosis patients

**DOI:** 10.1038/s41598-018-25992-7

**Published:** 2018-05-16

**Authors:** Federica Murgia, Silvia Svegliati, Simone Poddighe, Milena Lussu, Aldo Manzin, Tatiana Spadoni, Colomba Fischetti, Armando Gabrielli, Luigi Atzori

**Affiliations:** 1 Dipartimento di Scienze Biomediche, Università di Cagliari, Cagliari, Italy; 2 Dipartimento di Scienze Cliniche e Molecolari, Clinica Medica, Università Politecnica delle Marche, Ancona, Italy; 3 Unité de Chimie Environnementale et Interactions sur le Vivant, Université du Littoral Côte d’Opale, Dunkerque, France

## Abstract

Systemic sclerosis (SSc) is an autoimmune disease of unknown aetiology characterized by vascular lesions, immunological alterations and diffuse fibrosis of the skin and internal organs. Since recent evidence suggests that there is a link between metabolomics and immune mediated disease, serum metabolic profile of SSc patients and healthy controls was investigated by ^1^H-NMR and GC-MS techniques. The results indicated a lower level of aspartate, alanine, choline, glutamate, and glutarate in SSc patients compared with healthy controls. Moreover, comparing patients affected by limited SSc (lcSSc) and diffuse SSc (dcSSc), 6 discriminant metabolites were identified. The multivariate analysis performed using all the metabolites significantly different revealed glycolysis, gluconeogenesis, energetic pathways, glutamate metabolism, degradation of ketone bodies and pyruvate metabolism as the most important networks. Aspartate, alanine and citrate yielded a high area under receiver-operating characteristic (ROC) curves (AUC of 0.81; CI 0.726–0.93) for discriminating SSc patients from controls, whereas ROC curve generated with acetate, fructose, glutamate, glutamine, glycerol and glutarate (AUC of 0.84; CI 0.7–0.98) discriminated between lcSSc and dcSSc. These results indicated that serum NMR-based metabolomics profiling method is sensitive and specific enough to distinguish SSc from healthy controls and provided a feasible diagnostic tool for the diagnosis and classification of the disease.

## Introduction

Systemic sclerosis (scleroderma; SSc) is a systemic autoimmune disease characterized by vascular lesions, immunological alterations and diffuse fibrosis of the skin and internal organs caused by an increased deposition of extracellular matrix proteins^1^. The disease is highly heterogeneous and marked by a variable and unpredictable course. At the moment the etiopathogenesis of SSc is not defined yet and the absence of a clear picture about the pathogenic mechanisms leading to SSc has negative consequences both on early diagnosis and therapy. Diagnosis is usually late, and this impairs the effectiveness of available treatments. SSc can be clinically sub-classified based on patterns of skin fibrosis into limited (lcSSc) and diffuse (dcSSc) forms, that differ mainly in regards to the extent of cutaneous involvement. In lcSSc skin fibrosis is restricted to the fingers, distal extremities and face, whereas in dcSSc the trunk and visceral organs are also affected.

Regarding the pathogenetic mechanisms described in SSc patients or in experimental models of the disease, the vascular lesions with the ischemia-reperfusion events, the abnormal oxidative stress, the activation of the immune system, the gastrointestinal involvement often associated with small intestine bacterial overgrowth and malabsorption, are those with a relevant impact on metabolic processes^2–6^. However, aside from clinical studies documenting malnutrition in a high percentage of SSc patients and its correlation with disease severity and mortality, metabolic abnormalities have not been subjected to deep investigation.

Thus, metabolomics may be instrumental for the identification of potential biomarkers^7^, analysis of pathogenic mechanisms^8^, monitoring therapeutic responses^9^, drug discovery process^10^, role of the nutrition^11^, and effects of the environment^12^. Similar to genomics and proteomics, which carry the resulting information from the expression of genes and proteins, metabolomics refers to the whole metabolic profile of the cells or organism by studying systematically the effect of various biological factors on the metabolome. Metabolomics represents the possibility to quantify and identify low molecular weight metabolites in the same experiment which, as the final products of physiopathological processes, may be used for a better understanding of upstream biological events^13^. Metabolomics methods offer an integrated “snapshot” of the metabolic change during different conditions such as physiological status, diseases, exposure to drugs^14–16^ combining the use of several technologies, *e*.*g*. mass spectrometry (MS)^17^ and nuclear magnetic resonance (NMR) spectroscopy^18^, with pattern recognition techniques^19,20^. Mass-spectrometry in combination with separation techniques such as gas chromatography (GC) and liquid chromatography (LC) have been used in numerous metabolomics investigations^21,22^. MS is a highly sensitivity technique that allows detection of many metabolites in complex biological samples. NMR is a fast and simple instrumental platform for the metabolic analysis of biofluid, with high reproducibility and the ability to simultaneously quantify multiple classes of metabolites.

Recent studies evaluated the metabolic fingerprint of autoimmune diseases like multiple sclerosis, systemic lupus erythematosus and rheumatoid arthritis^23–26^. Since there are no complete metabolomics studies of SSc patients, we combined mass spectrometry gas chromatography (GC-MS) and ^1^H-NMR to identify a distinctive signature in serum sample from SSc patients that could be a useful approach to better understand the pathogenesis of the disease and to provide new biomarkers for scleroderma.

## Results

### Characteristics of the study subjects

Demographic, clinical and immunological features of the enrolled patients are summarized in Table 1. SSc patients had a mean age of 58.7 (22 to 81) years old and a mean disease duration (defined as the time from the onset of the first non-Raynaud’s phenomenon clinical manifestation) of 8 years. Twenty nine patients were female, and Caucasian. Twenty three had limited cutaneous SSc (lSSc) and fourteen patients had diffuse cutaneous SSc (dSSc).

**Table 1:** Demographic and clinical features of patients with systemic sclerosis and healthy controls.

	SSc (n = 37)	HC (n = 20)
Female/Male	29/8	15/5
Age (Range)	58.7 (22–81)	52 (30–68)
Disease Duration in years* (Range)	8 (1–19)	—
Anti- DNA Topoisomerase I/ACA, n (%)	11 (34%)/10 (31%)	—
lcSSc/dcSSc, n (%)	23 (62%)/14 (38%)	—
Visceral organ involvement		
Skin	29 (90%)	—
ILD	21 (65%)	—
GI	17 (53%)	—

*The time from the onset of the first non-Raynaud’s phenomenon clinical manifestation.

### ^1^H-NMR analysis and GC-MS analysis

Representative ^1^H-NMR spectra (Supplementary Fig. S1) and the corresponding table with the identified metabolites (Supplementary Table S1) and GC-MS chromatogram (Supplementary Fig. S2) of the analyzed serum are shown in the supplemental material.

**Figure 1:**
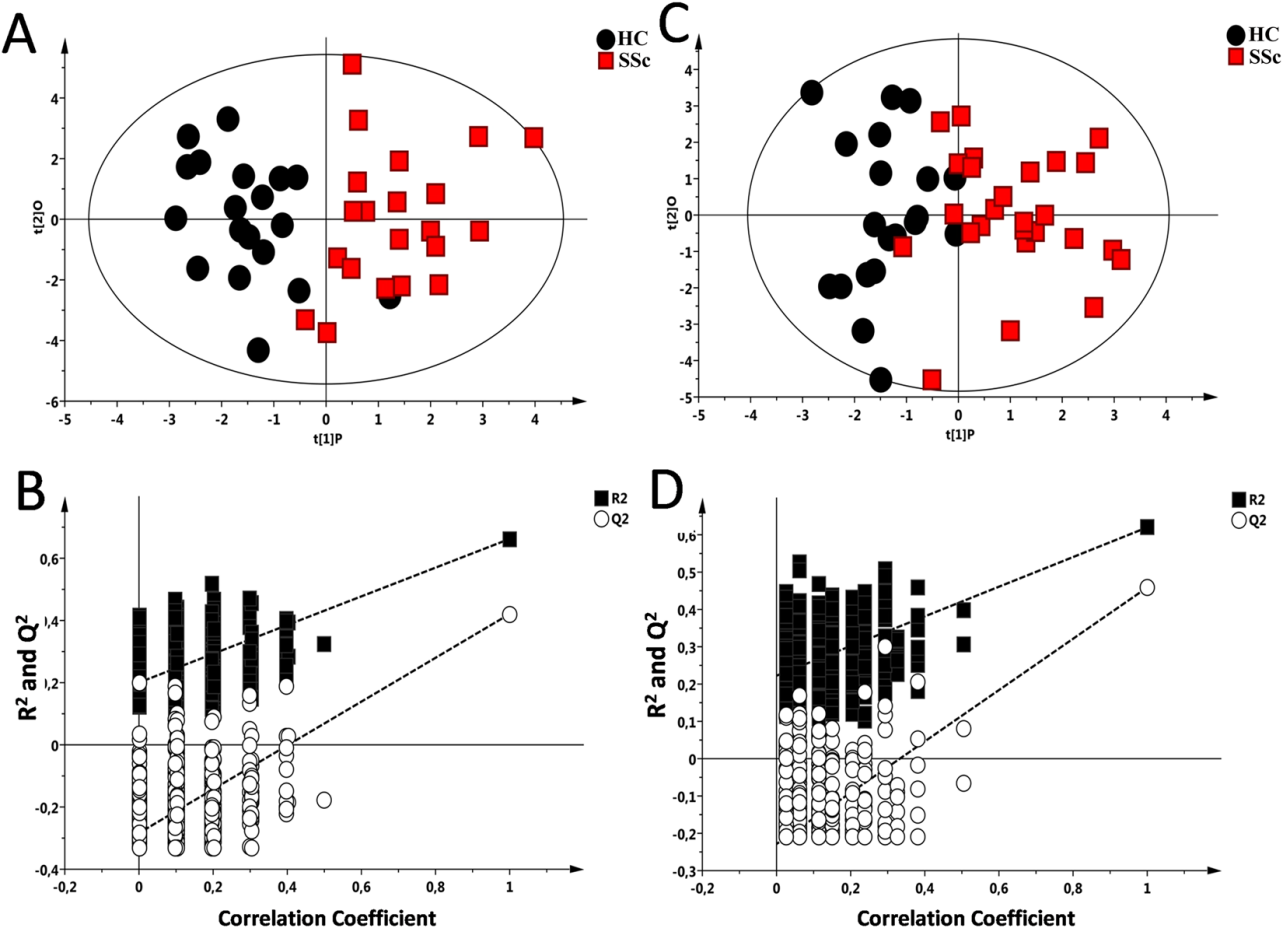
^1^H-NMR analysis and GC-MS analysis of serum Healthy Control (HC) and Systemic Sclerosis (SSc) samples. (**A**) Supervised model OPLS-DA score plot (R^2^X = 0.784, R^2^Y = 0.662, Q^2^ = 0.5; p < 0.0001) obtained from NMR spectra of serum samples from HC subjects and SSc patients (**B**) Validation of the model via permutation test (n = 400). (**C**) Supervised model OPLS-DA score plot (R^2^X = 0.38, R^2^Y = 0.620, Q^2^ = 0.464; p < 0.001) from HC and SSc samples analysed with the GC-MS, (**D**) Validation of the model via permutation test (n = 400).

Serum samples from SSc patients and HC were analyzed with the ^1^H-NMR. The Principal Component Analysis (PCA) was performed on the bins dataset of the samples to verify the presence of outliers that could affect the model (Supplementary Fig. S3). The Hotelling’s T2 test identified 9 strong outlier in the SSc group, which were then discarded. At the end, a total of 48 samples were used for the subsequent analyses. Afterwards Orthogonal-Partial Least Square-Discriminant Analysis (OPLS-DA) model was performed matching patients for age and sex (HC n = 20, female/male 15/5, age/range 52/30–68, SSc n = 28, female/male 22/6, age/range 56.3/28–74) revealing clear separation of HC from SSc group (R^2^X = 0.784, R^2^Y = 0.662, Q^2^ = 0.5 p < 0.001) (Fig. 1A and B).

The analysis of the GC-MS chromatograms was conducted on the same samples. The PCA model was performed using the matrix containing the normalized relative concentration of metabolites (Supplementary Fig. S3). Three samples (1 HC and 2 SSc) resulting strong outliers by Hotelling’s T2 test were excluded by the analysis. The OPLS-DA model was used to elucidate the most reliable class-discriminating variables using the concentrations of 16 metabolites having a VIP value >1 (Fig. 1C and D). In the OPLS-DA model the parameters of R^2^Y and Q^2^ were respectively 0.620 and 0.464 and p < 0.0001. These results revealed the good discrimination and predictive capability of this model.

**Figure 2:**
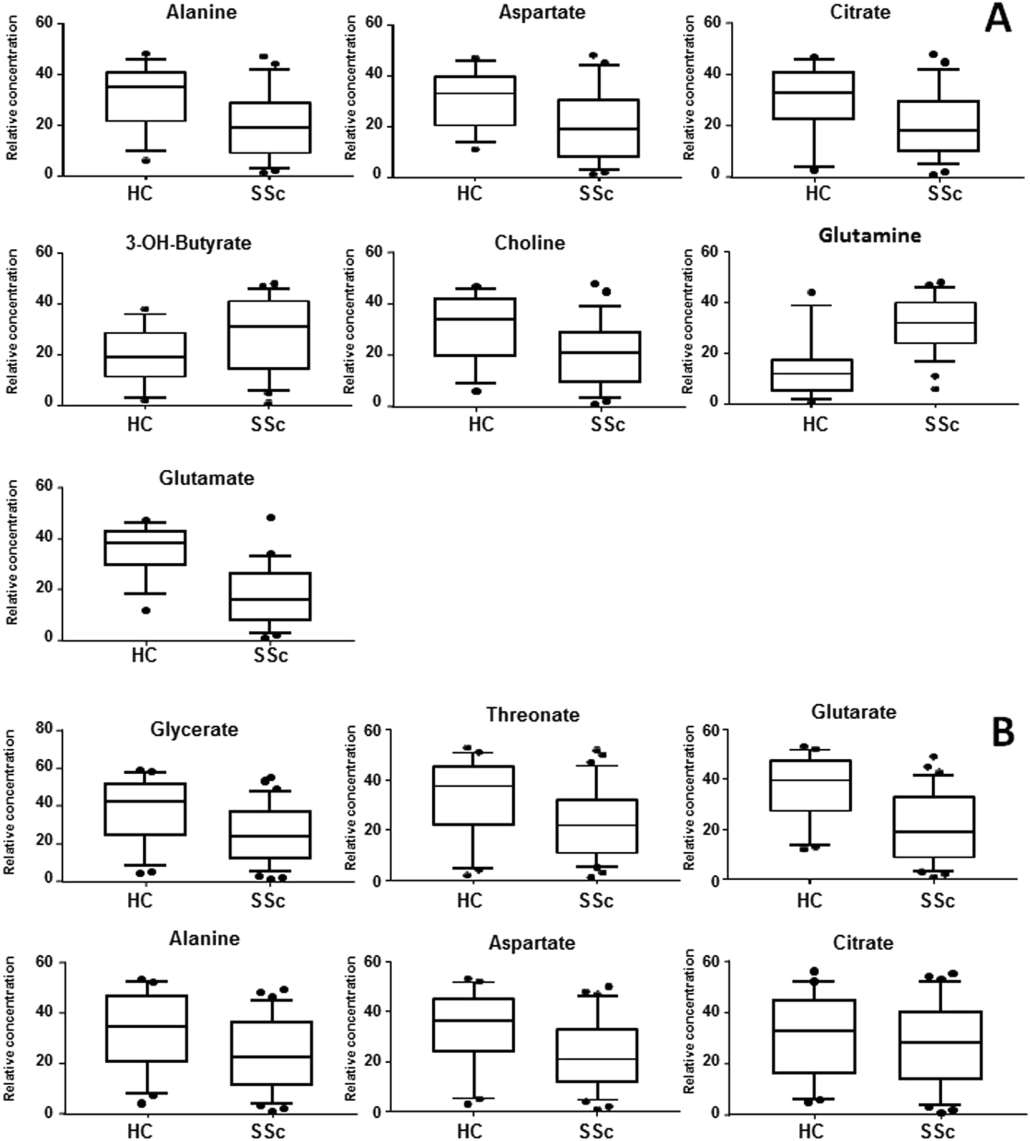
Box-plot of the discriminant metabolites in HC and SSc samples. (**A**) Box-plot of the metabolites significantly different (p < 0.05) in HC and SSc serum samples from the NMR analysis. (**B**) Box-plot of the metabolites significantly different (p < 0.05) between HC and SSc from the GC-MS analysis.

The list of the most discriminant metabolites resulting from the statistical models, both with ^1^H-NMR and GC-MS analysis, is reported in Table 2. All the discriminant metabolites underwent the U-Mann Whitney test and Holm-Bonferroni correction for p values. The results indicated a significant decreased level of aspartate, alanine, choline, glutamate and glutarate and an increased concentration of glutamine in the SSc compared to healthy control group. The box-plots of the metabolites concentration with p < 0.05 are showed in Fig. 2.

**Table 2:** Summary of the most important metabolites detected with ^1^H-NMR and GC-MS resulting from the analysis between HC and SSc and between dcSSc and lcSSc.

	Metabolites Model HC vs SSc	HC	SSc	Metabolites Model dcSSc vs lcSSc	dcSSc	lcSSc
^1^H-NMR	Acetate	+	−	Acetate	+	−
	Glutamate	+	−	Fructose	+	−
	Dimethylurate	+	−	Glutamate	+	−
	Lysine	+	−	Glycerol	+	−
	3-OH-butyrate	−	+	Lysine	+	−
	Lactate	−	+	Valine	+	−
				Lactate	−	+
				Glutamine	−	+
GC-MS ^1^H-NMR	Alanine	+	−	Sugars	−	+
	Aspartic acid	+	−			
	Citric Acid	+	−			
	Sugars	−	+			
GC-MS	2-pyrrolidone	−	+	Sorbitol	−	+
	D-threitol	−	+	Glycerate	+	−
	Butanoic Acid	−	+	Glutarate	+	−
	Glutaric Acid	+	−			
	L-threonic Acid	+	−			
	1–5-anhydrosorbitol	+	−

**Figure 3:**
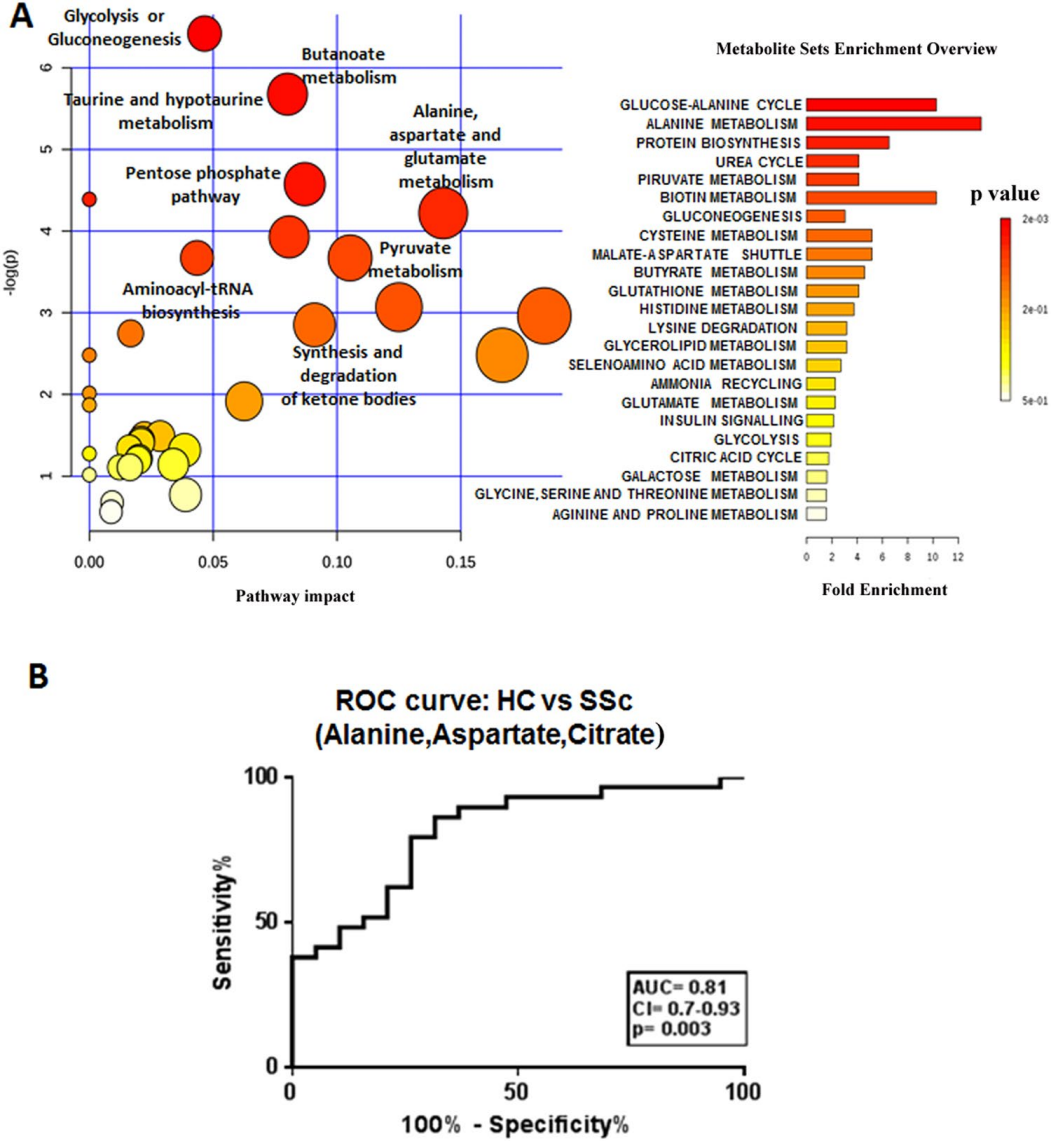
Metabolic pathway analysis of HC and SSc samples. (**A**) Summary of pathway analysis of SSc group compared to HC class with MetaboAnalyst 3.0. X axis represents the impact of the identified metabolites on the indicated pathway. Y axis indicates the extent to which the designated pathway is enriched in the identified metabolites. Circle colours indicate pathway enrichment significance. Circle size indicates pathway impact. (**B**) ROC curve generated with the relative concentrations of discriminant metabolites (alanine, aspartate and citrate) that were significantly different between HC and SSc both by NMR and GC-MS analysis.

The pathways analysis, performed using all the significantly different metabolites resulting from the multivariate analysis, underlined glycolysis or gluconeogenesis, butanoate metabolism, alanine, aspartate and glutamate metabolism, taurine and hypotaurine metabolism and energetic pathways like, pentose phosphate metabolism, synthesis and degradation of ketone bodies and pyruvate metabolism as the most important networks (Fig. 3A).

To evaluate the presence of possible diagnostic biomarkers, the predictive capability of the discriminant metabolites common in both ^1^H-NMR and MS techniques (aspartate, alanine and citrate) was tested through the ROC curve analysis. As demonstrated by statistical parameters of the ROC curve (AUC: 0.81, CI 07–0.93) these metabolites showed a good prognostic power (Fig. 3B).

**Figure 4:**
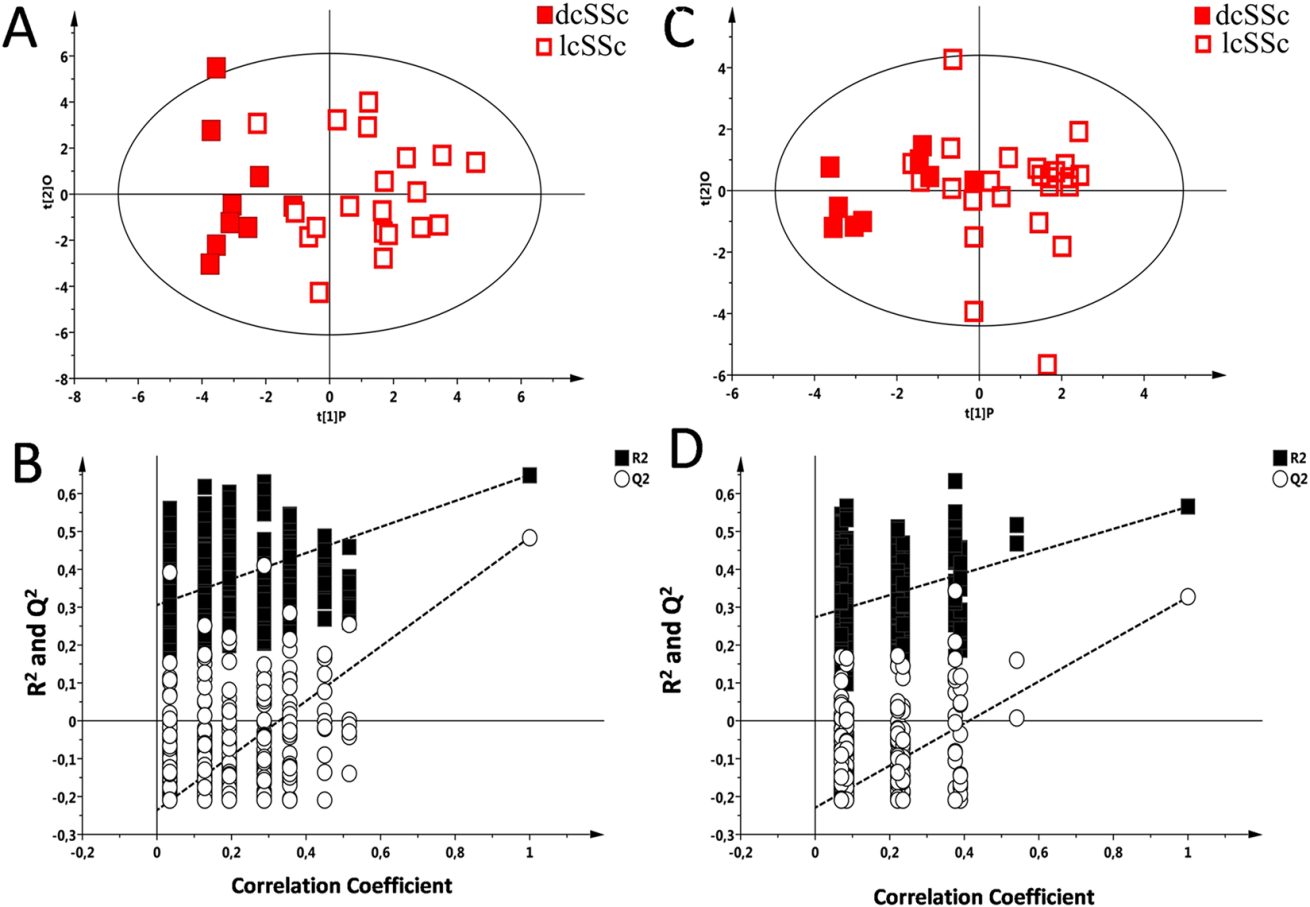
^1^H-NMR analysis and GC-MS analysis of diffuse cutaneous SSc (dcSSc) and limited cutaneous (lcSSc) samples. (**A**) Supervised model OPLS-DA score plot (R^2^X = 0.553, R^2^Y = 0.649, Q^2^ = 0.501, P < 0.001) generated using the NMR serum spectra metabolites of serum samples from dcSSc and lcSSc patient group. (**B**). Validation of the model via permutation test (n = 400). (**C**) Supervised model OPLS-DA score plot (R^2^X = 0.459, R^2^Y = 0.565, Q^2^ = 0.323; p < 0.01) from dcSSc and lcSSc samples based on GC-MS spectra. (**D**) Validation of the model via permutation test (n = 400).

In a second step, patients with dcSSc and patients with lcSSc were compared. The list of the most discriminant metabolites resulting from the statistical models is reported in Table 2. The score plot of the OPLS-DA model, based on ^1^H-NMR analysis, highlighted two clusters corresponding to dcSSc and lcSSc groups (Fig. 4A and B) with a R^2^Y = 0.649 and Q^2^ = 0.501 and a p < 0.001.

The OPLS-DA model, obtained from the GC-MS analysis, comparing dcSSc *vs* lcSSc demonstrated a clear separation between the two classes (Fig. 4C and D) with a Q^2^ = 0.323 and an R^2^Y = 0.565. The statistical value returned by CV-ANOVA was p < 0.01.

The ^1^H-NMR analysis identified an increased levels of valine, acetate, fructose, glutamate, glycerol, and lysine in the dcSSc class, while lcSSc patients showed enhanced concentrations of sugars, lactate, glutamine. The spectra obtained by GC-MS analysis, indicated a decreased presence of sugars and sorbitol and an increased concentration of glycerate and glutarate in the dcSSc samples compared to lcSSc.

**Figure 5:**
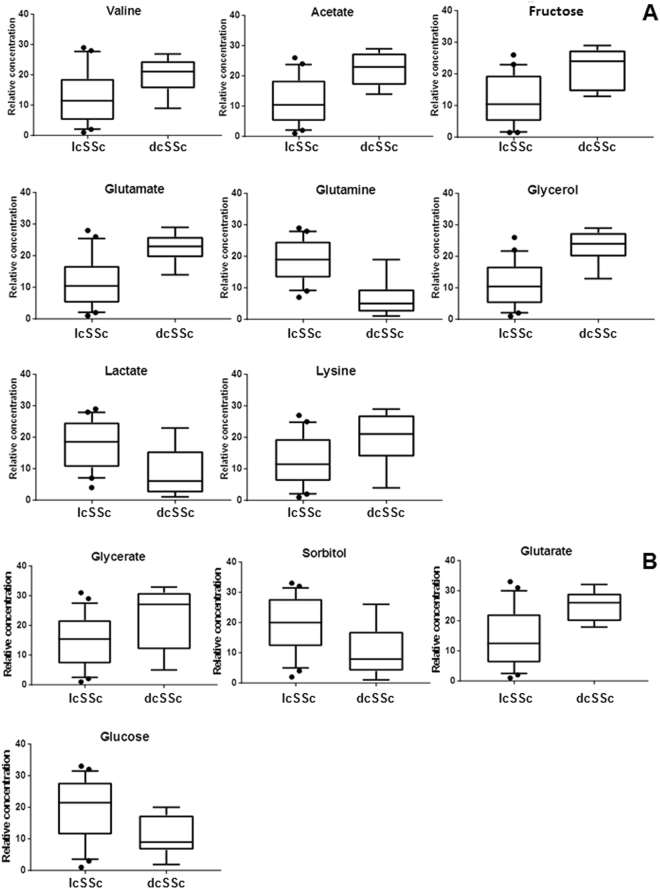
Box-plot of the discriminant metabolites in diffuse cutaneous SSc (dcSSc) and limited cutaneous SSc (lcSSc) samples. (**A**) Box-plot of the metabolites significantly different (p < 0.05) between dcSSc and lcSSc from the NMR analysis. (**B**) Box-plot of the metabolites significantly different (p < 0.05) between dcSSc and lcSSc from the GC-MS analysis.

**Figure 6:**
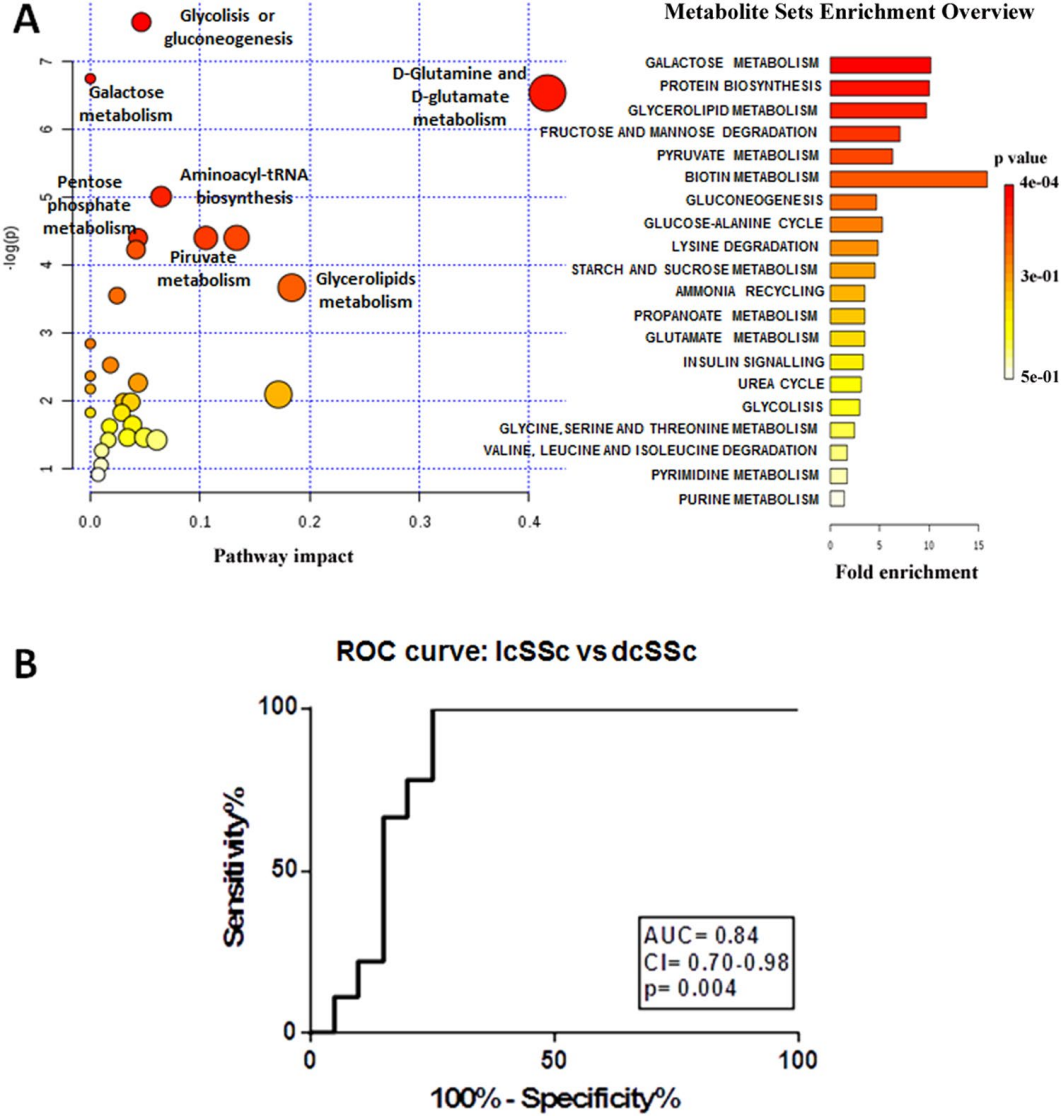
Metabolic pathway analysis of diffuse cutaneous (dcSSc) and limited cutaneous (lcSSc) samples. (**A**) MetaboAnalyst 3.0 pathway analysis in dcSSc vs lcSSc. X axis represents the impact of the identified metabolites on the indicated pathway. Y axis indicates the extent to which the designated pathway is enriched in the identified metabolites. Circle colors indicate pathway enrichment significance. Circle size indicates pathway impact. (**B**) ROC curve generated with the metabolites significantly different between dcSSc vs lcSSc that passed the Holm-Bonferroni test correction.

The box-plots of the metabolites resulting discriminant from the statistical analysis and with a p value < 0.05 obtained from the U-Mann Whitney test are represented in Fig. 5. The pathway analysis performed using Mataboanalyst 3.0, considering all significantly different metabolites, revealed glutamine and glutamate metabolism, glycolysis, gluconeogenesis and pathways involved in the energy production as the most important pathways. (Fig. 6A).

All metabolites passing the Holm-Bonferroni correction (*e*.*g*. acetate, fructose, glutamate, glutamine, glycerol and glutarate) were used to generate the ROC curve (AUC: 0.84, CI 0.7–0.98) (Fig. 6B). These statistical findings confirmed the diagnostic performance of the model by classifying the patients in the two studied groups.

## Discussion

Systemic sclerosis is characterized by heterogeneous clinical manifestations and is anything but simple to have a clear view of its clinical and physiopathology feature Scleroderma is devoid of reliable biomarkers predicting disease severity and progression; therefore, early diagnosis and disease monitoring are largely inadequate Aim of our study was to define possible metabolic changes in patients affected by SSc using gas-chromatography mass spectrometry and high resolution ^1^H-NMR spectroscopy and discover new potential biomarkers for the diagnosis of the pathology and a more accurate classification of the disease subsets. We identified a total of 13 discriminant metabolites significantly different between HC group and SSc group, and several altered metabolic pathways. Citrate content was significantly decreased in serum of SSc patients compared to controls. Citrate is a crucial molecule in the metabolism of energy production^27^. After its synthesis in the mitochondria, this metabolite could be employed in the Krebs cycle to promote the oxidative phosphorylation with a resulting ATP production^28^. Under inflammatory condition, as observed in the case of systemic lupus erythematosus^29^, the decreased energy availability increases its demand. The altered blood level of the intermediates of the Krebs cycle, like the citric acid, in SSc samples could be explained by the high consumption to supply the energy deficiency. In addition to its primary role in the cellular energy production, citric acid has been demonstrated to have important immunologic properties, modulating the pro-inflammatory processes in macrophages^27^. Furthermore, in line with our finding, several studies reported a decreased level of citric acid in urine and serum of patients affected by immune-mediate disease^30–32^. In particular, Alonso *et al*. detected lower level of citrate in patients affected by psoriasis and SLE underlining a key-role of this metabolite in immune-mediated inflammatory diseases^26^.

Similarly, the low level of L-alanine, and L-lysine found in SSc patients may be ascribed to the metabolites employment in the regulation of inflammatory and autoimmune response^33^.

Aspartate was another interesting metabolite that we found decreased in the SSc patients. Aspartate plays an important role in the metabolism and functions of leucocytes. Moreover, as a substrate for the synthesis of purine and pyrimidine nucleotides, aspartate is crucial for the proliferation of lymphocytes^33^.

The metabolic pathway analysis identify some relevant metabolic nets mainly including glycolysis, gluconeogenesis, pathways related to the energetic metabolism, butanoate metabolism, taurine and hypotaurine metabolism and alanine, aspartate and glutamate metabolism. The pathways identified as relevant in our models, have been previously reported to play a pivotal role in resting or activated T cells^34^.

Bioenergetic metabolism is necessary for the cells of the immune system to carry out its specific functions^35^. In autoimmune diseases a chronic immune activation may exceed the physiological bioenergetics metabolism. For this reason, in immunological and other chronic inflammatory diseases (es. rheumatic disease and SSc), the activation of the immune system consumes considerable amount of energy (up to 2,000-kJ/day and more)^36^. Moreover, it has been shown that, in response to cellular changes of glucose levels, glycolysis and gluconeogenesis pathways are strictly and mutually regulated^37^. Control of glycolysis and gluconeogenesis seems to be lost in autoimmune disease as a consequence of the infiltration by the immune cells of tissues causing a relative decrease of the oxygen/cell ratio. Moreover accumulation of inflammatory cell and the oedema increase the distance between cells and oxygen-supplying arterial vessels. In addition the vasodilatation, induced by inflammatory processes, lowers the blood flow and the consequent supply of oxygen and nutrients^38–40^. Glycolysis has been suggested to play a role in the pathogenesis of rheumatoid arthritis^41^ and the activity levels of two major enzymes of the glycolytic pathway – glyceraldehydes 3-phosphate dehydrogenase and lactate dehydrogenase – were increased in the synovial cells^42^. Interestingly, keloid “scar” fibroblasts, which share many characteristics with SSc fibroblasts, are known to use aerobic glycolysis as their primary energy source^43^. In an experimental model of SSc, the role of glycolysis as an important mechanism to assure the energy efficiency of the fibroblasts has ben shown^44^.

At the moment, studies of a direct relationship between increased glycolytic activity and inflammation are lacking. However, glycolytic inhibitors may be consider a new therapeutic strategy for preventing fibrosis in scleroderma patients. Bonnet *et al*. demonstrated that a glycolytic inhibitor reduced fibrosis and increased survival in fawn-hooded rats^45^.

Since clinical presentation in patients with SSc is highly variable, disease subgrouping as well as prediction of future organ involvement and prognosis are extremely important for the clinical setting. Metabolomics may have the potential to be used as a measure of disease severity in SSc, considering the ability to partly distinguish the less severe SSc patients. The most important pathways altered in diffused SSc compared to limited SSc were glycolysis and gluconeogenesis, pathways related to the energetic metabolism and glutamate-glutamine metabolism. In addition to glucose, amino acids are have an important role for T cells as a prime source of energy and as a substrate for protein and nucleic acid biosynthesis^34^. Glutamate is a substrate for the synthesis of γ-aminobutyrate (GABA), which is present in both lymphocytes and macrophages. T cells have a functional GABAergic system, which is involved in T-cell immune response^46^. Moreover, glutamate, an immediate precursor for glutathione, plays a regulatory role in its synthesis, removing oxidants^47,48^. Glutamine, increased in patients with lcSSc, is a relevant energy substrate for cells of the immune system^49^, playing an important role in their function and homeostasis. Glutamine, but not glutamate, uptake is enhanced during T-cell activation^50^. This may explain the increase of glutamate and the decrease of glutamine in patients with dcSSc compared to lcSSc patients. In addition to be essential for protein synthesis, glutamine contributes to other processes important for proliferating T cells, including fatty acid synthesis, synthesis of purine and pyrimidine nucleotides.

In line with our results recent studies on the metabolic fingerprint of inflammatory disease based on an autoimmune process, such as rheumatoid arthritis, psoriatic arthritis, psoriasis, systemic lupus erythematosus, Crohn’s disease, and ulcerative colitis, alanine and citrate were identified as discriminant metabolites^26^. This finding suggest a non-specific role for alanine and citrate in SSc. On the contrary aspartate has been found to be discriminant in systemic sclerosis but not in the other inflammatory and autoimmune disorders^26^.

In conclusion, our metabolomic approach allowed the identification of significant biological molecules that are discriminant between SSc and HC and the resulted pathways involved. This may be useful to better clarify the pathophysiology of SSc and for the classification of the patients in the different subtypes of scleroderma. This study represents a preliminary step for future largest study. Further investigations with a wider cohort of patients are necessary to elucidate the significance of the observed changes.

## Materials and Methods

### Patients

Thirty-seven SSc Caucasian patients were enrolled for the study. All patients fulfilled the diagnosis of systemic sclerosis according to the ACR/EULAR 2013 criteria^51^ and were classified in limited and diffuse cutaneous SSc (lcSSc and dcSSc, respectively) as described^51^. Inclusion criteria were: SSc, as defined above, age 18 to 80 years and ability to give informed consent. Pregnancy had to be ruled out before the beginning of the study. Exclusion criteria were the presence of an infectious diseases at study entry and the use of steroids or immunosuppressive drugs in the previous 6 months. Demographic, clinical, and immunological features of the enrolled patients are summarized in Table 1.

Study protocol, patient information sheet and consent form were all approved by the local Ethics Committee of Marche Region, Italy. The study was conducted in accordance with the Declaration of Helsinki, V edition (2000). Written informed consent was obtained from all patients and controls.

Control sera (20 samples) were obtained from age, sex, and race-matched normal, nonsmoking, healthy volunteers. They were selected according to the following inclusion criteria: good health, no clinical history of autoimmune disease, free from medical therapy.

### Sample preparation

The serum samples were extracted as previously described^52^. Briefly, serum samples were thawed and centrifuged at 2500 g for 10 min at 4 °C. An 800 µl aliquot was added to 2400 µl of a chloroform/methanol 1:1 plus 350 µl of distilled water. The samples were vortexed for 1 min and centrifuged for 30 min at 1700 *g* at room temperature. The hydrophilic and hydrophobic phases were obtained. The water-phase was divided in 2 aliquots, concentrated overnight using a speed vacuum centrifuge for GC-MS and ^1^H-NMR analysis.

### ^1^H NMR sample preparation and analysis

1400 ul of the water-phase for each sample was concentrated overnight in a speed-vacuum. The concentrated water-phase was resuspended in 630 µl of D2O and 70 µl trimethylsilyl propanoic acid (TSP) 5.07 mM. TSP was added to provide an internal reference for the chemical shifts (0 ppm), and 650 µl of the solution were transferred to a 5 mm NMR tube.

The samples were analyzed with a Varian UNITY INOVA 500 spectrometer (Agilent Technologies, Inc., Santa Clara, CA, USA), which was operated at 499 MHz equipped with a 5 mm triple resonance probe with z-axis pulsed field gradients and an auto-sampler with 50 locations. One-dimensional ^1^H-NMR spectra were collected at 300 K with a pre-sat pulse sequence to suppress the residual water’s signal. The spectra were recorded with a spectral width of 6000; a frequency of 2 Hz; an acquisition time of 1.5 s; a relaxation delay of 2 ms; and a 90° pulse of 9.2 µs. The number of scans was 256. Each Free Induction Decay (FID) was zero-filled to 64 k points and multiplied by a 0.5 Hz exponential line-broadening function. The spectra were manually phased and baseline corrected. By using MestReNova software (version 8.1, Mestrelab Research S.L.) each NMR spectrum was divided into consecutive “bins” of 0.04 ppm. The spectral area investigated was the region between 0.6 and 8.6 ppm. The regions between 4.60 and 5.2 ppm and between 5.24 and 6.6 ppm were excluded to remove variations in the pre-saturation of the residual water resonance and spectral regions of noise. To minimize the effects of the different concentrations of serum samples, the integrated area within each bin was normalized to a constant sum of 100. The final data set consisted of a 146 × 57 matrix. The columns represent the normalized area of each bin (variables), and the rows represent the samples (subjects).

### GC-MS sample preparation and analysis

700 µL of the water-phase for each sample was concentrated overnight in a speed-vacuum. Blanks were made following the same procedure used for the samples to avoid noises due to the chemicals used for the preparation and the laboratory instruments.

Derivatization was made adding to dried samples 100 μL of methoxyamine hydrochloride in pyridine solution (10 mg/mL) for 17 hr. Subsequently, 100 μL of N-trimethylsilyltrifluoroacetamide (MSTFA) were added and vortexed at R.T., 1 h. Samples were then diluted in hexane (600 μL) with an internal standard (undecane at 25 ppm). Diluted samples were then filtered (PTFE 0.45 μm) and transferred in glass vials.

A 1 μL aliquots of the samples was injected splitless by an autosampler in a Agilent 7890 A gas chromatograph coupled with an Agilent 5975 C mass spectrometer equipped with a HP-5MS capillary column (5%-Phenyl-methylpolysiloxane; 30 m, 25 mm i.d., 0.25 μm film thickness). The initial oven temperature was 50 °C (hold 3 min) and increased at 10 °C/min to 250 °C for a total run of 35 min. Spectra were acquired in electron impact mode and full scan monitoring mode (m/z 50–800). The injector and ion source temperature were respectively set at 200 and 250 °C. Helium was used as carrier gas in constant pressure mode (7.6522 psi).

Trimethylsilated metabolites were identified via the injection of derivatized pure standards and checking the NIST library or matching the obtained spectra with those available on the Human Database Metabolome (HMDB) as previously described^23^.

### Statistical analysis

A multivariate statistical analysis was performed using SIMCA-P software (ver. 13.0, Umetrics, Sweden). The variables were Pareto scaled to emphasize all metabolite signals and reduce the spectral noise for the ^1^H-NMR analysis and UV scaled for the GC-MS analysis.

The initial data analyses were conducted using the Principal Component Analysis (PCA), which is important for the exploration of the sample distributions without classification. To identify potential outliers, the DmodX and Hotelling’s T2 tests were applied.

Orthogonal Partial Least Square (OPLS-DA) was subsequently applied. OPLS-DA maximize the discrimination between samples assigned to different classes. The variance and the predictive ability (R^2^X, R^2^Y, Q^2^) were established to evaluate the suitability of the models. OPLS-DA models were performed by using only bins corresponding to VIP (Variable Influence on Projection) value >1.

One can compare the VIP of one term to the others. Terms with VIP larger than 1 are the most relevant for explaining Y (assignment of two classes)^53^. In addition, a permutation test (n = 400) was performed to validate the models. The scores from each OPLS-DA model were subjected to a CV-ANOVA to test for significance (p < 0.05).

The most significant variables were extracted by the loading plot from each model and for the ^1^NMR data were identified using the Chenomx NMR Suite 7.1 (Chenomx Inc., Canada)^54^. GraphPad Prism software (version 7.01, GraphPad Software, Inc., CA, USA) was used to perform the univariate statistical analysis of the data. To verify the significance of the metabolites resulting from multivariate statistical analysis U-Mann Whitney test was performed. ROC curves were built to test the sensibility and the specificity of the pool of the selected metabolites with the same software.

### Pathways analysis

Metabolic pathways were generated by using MetaboAnalyst 3.0 (www.metaboanalyst.ca), a web server designed to obtain a comprehensive metabolomic data analysis, visualization and interpretation^55^. With this approach, it was possible to correlate metabolites changes with metabolic networks. In particular, the pathway analysis module of Metaboanalyst 3.0 combines results from powerful pathway enrichment analysis with the pathway topology analysis to help researchers identify the most relevant pathways involved in the study conditions. It uses the high-quality KEGG metabolic pathways as the backend knowledgebase.

All data generated or analysed during the current study are included in this article (and its Supplementary Information Files).

## Electronic supplementary material

Supplementary info
