# Supplementary material for: Metabolomic profile of systemic sclerosis patients
Source: Sci Rep. 2018 May 16;8:7626. doi: 10.1038/s41598-018-25992-7 (PMC5955890; doi:10.1038/s41598-018-25992-7)

Metabolomic profile of systemic sclerosis patients

Murgia F.[1], Svegliati S.[2], Poddighe S.[1,3], Lussu M.[1], Manzin A.[1], Spadoni T.[2], Fischetti C.[2], Gabrielli A.[2*], Atzori L.[1*]

[1] Dipartimento di Scienze Biomediche, Università di Cagliari, Cagliari, Italy.

[2] Dipartimento di Scienze Cliniche e Molecolari, Clinica Medica, Università Politecnica delle Marche, Ancona, Italy.

3 Unitè de Chimie Environnementale et Interactions sur le Vivant, Universitè du Littoral Côte d'Opale, Dunkerque, France

[*] Corresponding Author

Armando Gabrielli, MD

Dipartimento di Scienze Cliniche e Molecolari

Clinica Medica

Via Tronto 10,

60126 Ancona

Italy

phone:+39 071 2206104

Email: a.gabrielli@univpm.it

Luigi Atzori, MD

Dipartimento di Scienze Biomediche

Università di Cagliari,

Via Porcell 4

09124 Cagliari

Italy

latzori@unica.it

Supplementary Materials

Fig S1. Signal assignments of the serum metabolites in an NMR spectrum.

1. Lipid 1; 2. 2-OH-butyrate; 3. Isoleucine; 4. Leucine; 5. Valine; 6. 3-OH-butyrate; 7. Lipid 2; 8. Lactate; 9. Alanine; 10. Lysine; 11. Acetate; 12. Proline; 13. N-acetyl-groups; 14. Methionine; 15. Glutamate; 16. Pyroglutamate; 17. Glutamine; 18. Citrate; 19. Aspartate; 20. Sarcosine; 21. Asparagine; 22. Creatinine; 23. Creatine; 24.Ornithine; 25. Choline; 26. Glucose; 27. Betaine; 28. 1,3-Dimethylurate*; 29. Methanol**; 30. Glycine; 31. Glycerol; 32.Glucitol*; 33. Glycylproline*; 34. Fructose; 35. Threonine; 36. Tyrosine; 37. Histidine; 38. tryptophan; 39. Phenylalanine; 40. τ-methylhistidine; 41.Oxypurinol*: 42. Adenosine*; 43. Inosine*; 44. Formate. *** = uncertain attribution; ** = extraction contaminant.**

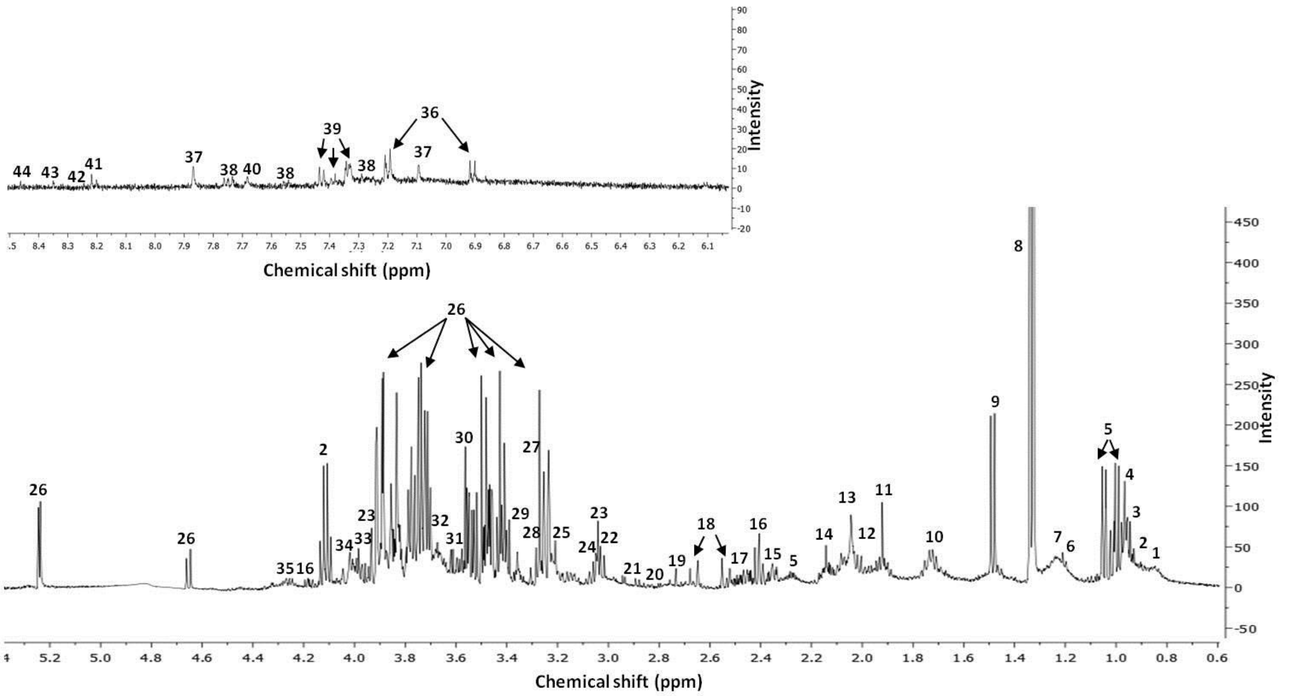

Table S1: Attributions of the metabolites identified in the NMR spectra with the corresponding chemical shifts.

**Compound Name**	**Chemical Shifts [± 0.03 ppm]**
1,3-Dimethylurate*	3.31; 3.43
2-Hydroxybutyrate	0.89; 1.64; 1.73; 3.98
3-Hydroxybutyrate	1.19; 2.30; 2.39; 4.14
Acetate	1.91
Adenosine*	8.33; 8.25; 6.06; 4.43; 4.29; 3.91; 3.83
Alanine	1.47; 3.78
Asparagine	2.85; 2.94; 4.00; 6.91; 7.62
Aspartate	2.68; 2.80; 3.89
Choline	3.20; 3.51; 4.06
Citrate	2.53; 2.69
Creatine	3.03; 3.92
Creatinine	3.03; 4.05
Formate	8.45
Fructose	3.55; 3.56; 3.59; 3.67; 3.70; 3.70; 3.79; 3.79; 3.82; 3,89; 3.99; 4.01; 4.10; 4.11
Glucitol*	3.63; 3.65; 3.72; 3.75; 3.77; 3.84
Glucose	3.24; 3.39; 3.40; 3.46; 3.48; 3.53; 3.70; 3.72; 3.76; 3.82; 3.84; 3.89; 4.64; 5.24
Glutamate	2.04; 2.12; 2.33; 2.36; 3.75
Glutamine	2.11; 2.14; 2.43; 2.46; 3.77; 6.87; 7.59
Glycerol	3.55; 3.65; 3.78
Glycine	3.55
Glycylproline*	1.97; 2.12; 2.24; 2.32; 3.53; 3.58; 3.95; 4.27;
Histidine	3.14; 3.24; 3.98; 7.09; 7.88
Inosine*	8.33; 8.23; 6.09; 4.43; 4.27; 3.91; 3.83
Isoleucine	0.93; 1.00; 1.25; 1.46; 1.97; 3.66
Lactate	1.32; 4.12
Leucine	0.94; 0.95; 1.67; 1.70; 1.73; 3.72
Lipid 1	0.82

Lipid 2	1.22
Lysine	1.43; 1.50; 1.72; 1.88; 1.91; 3.02; 3.75
Methanol**	3.36
Methionine	2.11; 2.13; 2.19; 2.63; 3.85
N-acetylgroups	2.02
Ornithine	1.74; 1.82; 1.93; 3.05; 3.78
Oxypurinol*	8.22
Phenylalanine	3.11; 3.27; 3.99; 7.32; 7.37; 7.42
Proline	1.98; 2.02; 2.34; 3.33; 3.41; 4.12
Pyroglutamate	2.02; 2.39; 2.50; 4.17
Sarcosine	2.74; 3.6
Threonine	1.32; 3.58; 4.25
Tryptophan	7.73; 7.53; 7.31; 7.27; 7.19; 4.05; 3.48; 3.30
Tyrosine	3.04; 3.19; 3.93; 6.89; 7.18
Valine	0.98; 1.03; 2.26; 3.60
τ-methylhistidine	7.76; 7.0; 3.96; 3.68; 3.16; 3.06

* = uncertain attribution; ** = extraction contaminant

Fig. S2 GC-MS chromatogram with the attributions of the metabolites in a serum sample

1. Lactic acid; 2 Valine; Retention Standard (R.S.); 3. 2-Hydroxybutyric acid; 4. Acetic acid; 5. 3-Hydroxybutyric acid; 6. Butanoic acid; 7. Urea; 8. Glycerol; 9. Phospahte; 10. Threonine;11. Glycine; 12. Glyceric acid; 13. Glutaric acid; 14. Aspartic acid; 15. 2-Pyrrolidone; 16. D-Threitol; 17. Glutamic acid; 18. Threonic acid; 19. Citric acid; 20. 1,5-Anhydro-Sorbitol; 21. Sugars; 22. Myo-inositol; 23. Oleamide.

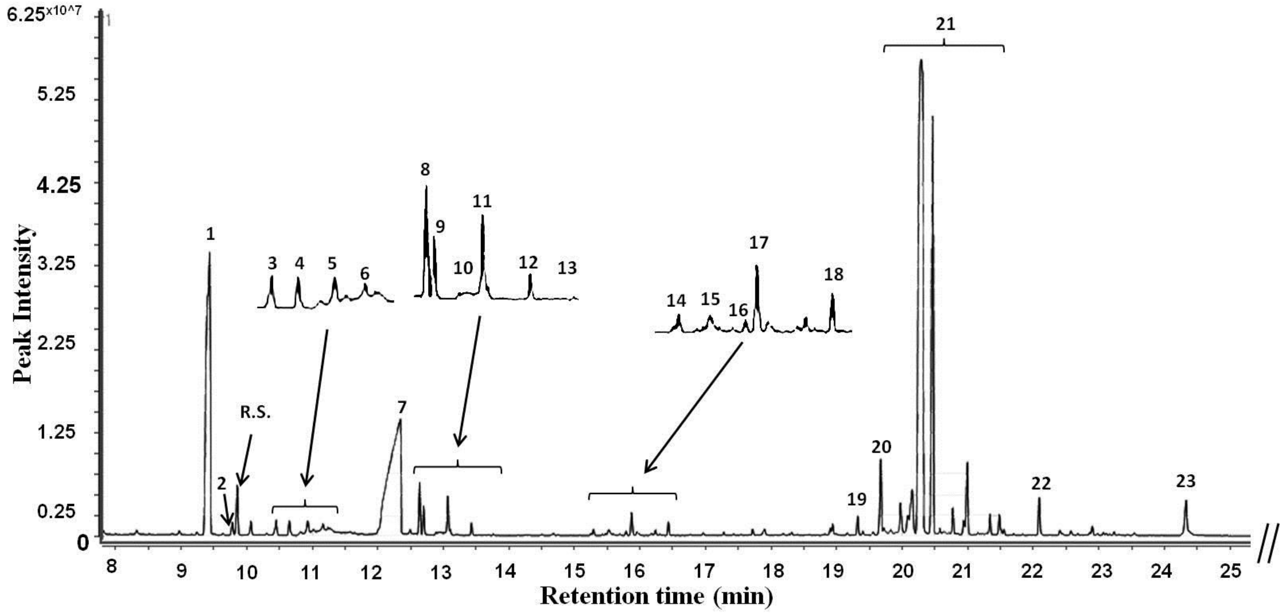

Fig. S3 PCA score plots resulting from the ^{1}H-NMR analysis and GC-MS analysis of Healthy Control (HC) and Systemic Scleroderma (SSc) samples, and of diffused cutaneous SSc (dcSSc) and limited cutaneous SSc (lcSSc) samples. A) PCA model of HC and SSc samples generated using the NMR serum spectra. B). PCA model of dcSSc and lcSSc samples generated using the NMR serum spectra C-D) PCA model of HC and and SSc samples generated using the GC-MS analysis. D. PCA model of dcSSc and lcSSc samples generated using the GC-MS analysis.

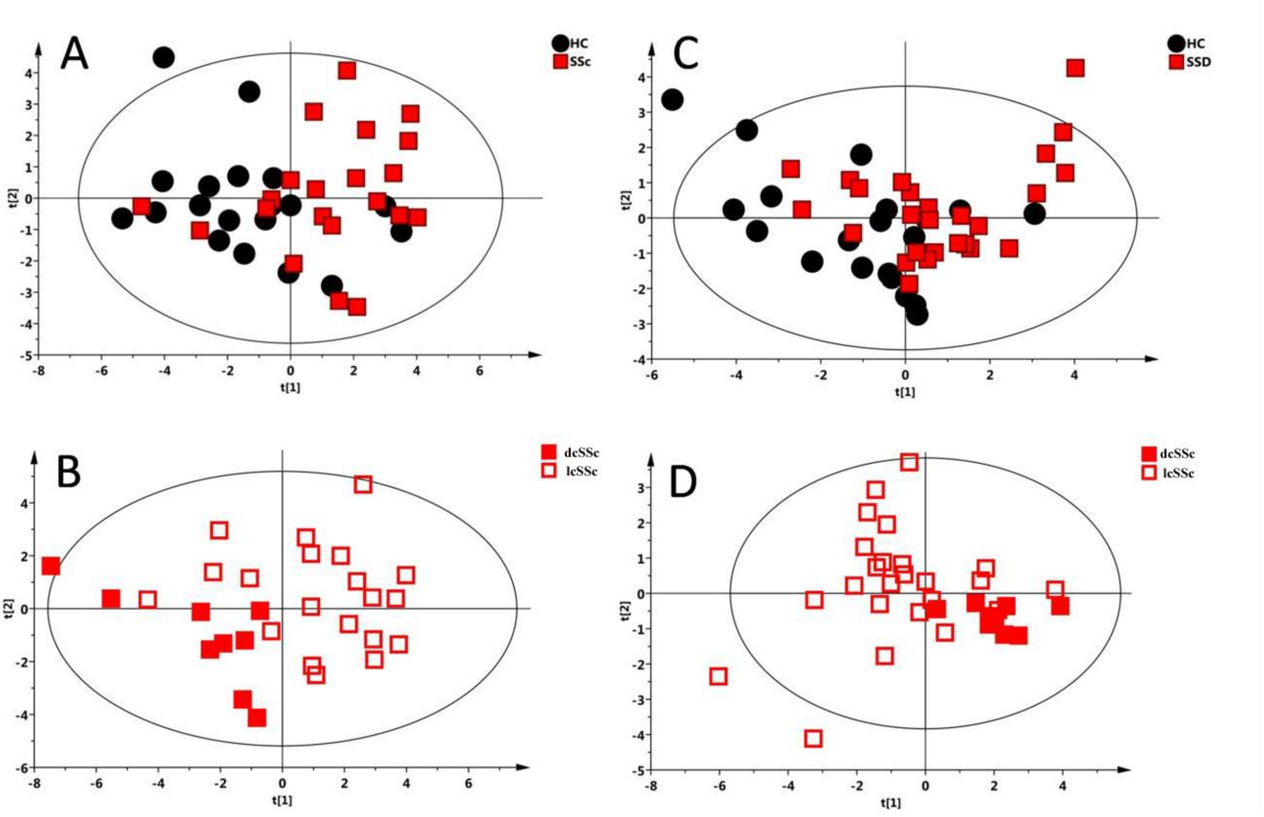

Supplement: Supplementary file 1 — Supplementary info [file 41598_2018_25992_MOESM1_ESM.pdf]
